# Editorial: Microorganisms and their metabolites as biocontrol agents for sustainable agriculture

**DOI:** 10.3389/fmicb.2022.1079016

**Published:** 2022-11-10

**Authors:** Gustavo Cordero-Bueso, Ileana Vigentini, Clemencia Chaves-López

**Affiliations:** ^1^Department of Biomedicine, Biotechnology and Public Health, University of Cádiz, Cádiz, Spain; ^2^Department of Biomedical, Surgical and Dental Sciences - One Health Unit, University of Milan, Milan, Italy; ^3^Faculty of Bioscience and Agri-Food Technologies, University of Teramo, Teramo, Italy

**Keywords:** crop plant pathogens, biocontrol agents, sustainable agriculture, fungal diseases, biofertilizers, biological control

Exposure to synthetic fertilizers or pesticides is linked to adverse health outcomes in humans and animals, and their overuse have also effects on the environment. Regulatory bodies of several countries have recognized the risks of pesticide exposure and are taking measures to drastically reduce their use. A large amount of fruit and vegetables loss occurs each year during pre and post-harvest stages due to plant pathogen infestation that involves a wide range of microorganisms (yeasts, bacteria, viruses, phytopathogenic fungi, and other microscopic parasites). Some antagonistic microorganisms have been postulated as less hazardous and equally effective substitutes for pesticides against a diverse population of plant pathogens. Flexible and scalable high-throughput technologies combined with other techniques are now available to understand how a future new generation of biocontrol agents can contribute to reduce crop diseases offering solutions to improve the sustainability and resilience of agricultural systems.

The Research Topic “*Microorganisms and their metabolites as biocontrol agents for sustainable agriculture*” belongs to the Microbe and Virus interactions with Plants section in the Frontiers in Microbiology journal. We present an overview of 8 published original research papers. Sui et al. reported an antagonistic bacterium identified as *Bacillus velezensis* EM-1 isolated from bacterial wilt-suppressive soil which could inhibit the growth of *Ralstonia solanacearum* in tobacco roots decreasing the occurrence of bacterial wilt disease. Moreover, EM-1 also exhibited a strong inhibitory effects in some phytopathogenic fungi such as *Phytophthora nicotianae* and *Alternaria alternata*, indicating a broad antagonistic spectrum. Furthermore, EM-1 can also activate plant resistance by increasing the activity of catalase and polyphenol oxidase enzymes in tobacco. This is in accordance with the study carried out by Li et al. which identified an endophytic bacterial strain of wild grape, *Bacillus subtilis* K1, as an excellent antagonist of *Botrytis cinerea* in *in vitro* and *in vivo* experiments. The *in vitro* experiments showed that K1 and its volatile organic compounds (VOCs) could significantly constraint the mycelia growth of *B. cinerea*. Indeed, grape fruit inoculated with *Bacillus* K1 displayed lower graymold proliferation in comparison to untreated samples. In particular, dibutyl phthalate was identified as the volatile substance with strong activity in suppressing the development of *B. cinerea* mycelium.

Other investigations carried out by Safara et al. revealed the presence of VOCs formed by the endophytic bacteria isolated from healthy sugar beet (*Beta vulgaris*) and sea beet (*Beta maritima*) belonging to the species: *Pantoea* sp. Dez632, *Pseudomonas* sp. Bt851, *Streptomyces* sp. B86, and *Stenotrophomonas* sp. Sh622. These VOCs were analyzed for their effects on the virulence traits of the bacterium strain *Bacillus pumilus* Isf19, the causal agent of the sugar beet root rot disease. Results of this study disclosed that VOCs produced by the strains Bt851 and Sh622 substantially reduced attachment of *B. pumilus* Isf19 cells to sugar beetroots. Furthermore, all endophytic bacteria tested drastically reduced chemotaxis motility of the pathogen toward root extract. Specifically, the VOCs produced by Dez632 and Bt851 upregulated the expression levels of defense genes related to soft rot resistance. Wu et al. showed for the first time that *B. velezensis* GJ-7 and *Bacillus cereus* NS-2 had the potential for fruitful ecological niche colonization and enhanced plant resistance, thus behaving as potential biocontrol agents against root-knot nematodes. The rhizosphere *B. velezensis* strain GJ-7 and *B. cereus* strain NS-2 showed high nematicidal and eggs activity against *Meloidogyne hapla*, reducing the sum of root galls in several *in vitro* control experiments. On the other hand, Wang et al. emphasized the importance of *Streptomyces lydicus* secondary metabolites and its potential application in agriculture. In this work, an liquid extract of *S. lydicus* strain M01 was used to treat *A. alternata* phytopathogen and morphological changes in the cell wall and plasma of hyphae and conidia were perceived. The results revealed more than one hundred twenty metabolites, mainly referred as, antibacterials, fungicides, insecticides, herbicides and plant growth regulators, such as indole-3-acetic acid (IAA). Chai et al. showed the importance of *Streptomyces griseorubiginosus* LJS06 in the management of cucumber anthracnose, caused by *Colletotrichum orbiculare*, by directly priming the plant defense system and inhibiting conidial function. Physiological and biochemical tests revealed that *S. griseorubiginosus* LJS06 could produce cellulase, amylase, chitinase, polyamines, protease, siderophores, and IAA. Thus, an extract of LJS06 (specifically SL06) was prepared and assayed for its efficacy against appressorium formation, conidial germination and anthracnose management and significantly reduced cucumber anthracnose severity.

The work of Botcazon et al. delved with amphiphilic lipid compounds from bacteria secretomes, suggested to replace chemical pesticides for harvest protection. These compounds were fengycins (FGs) produced by fermentation of *Bacillus subtilis* Bs2504 and rhamnolipids (RLs) produced by *Pseudomonas aeruginosa*. Their biocidal effects were evaluated on two *Sclerotiniaceae* fungi species responsible for pathogenicity in diverse plant species worldwide. Authors showed that different strains of *B.cinerea* and *Sclerotinia sclerotiorum* had opposite sensitivities to FGs and RLs on plate assays. *B. cinerea* was more sensitive to FGs while *S. sclerotiorum* was more sensitive to RLs. Contrarily, Kim et al. evidenced that 2,3-butanediol (BDO)-producing PGPR could control tomato bacterial wilt. They found that *Klebsiella pneumoniae* JCK-2201 produced elevated concentration of 2,3-BDO. The control ability and the mechanism of meso-2,3-BDO reached by the strain JCK-2201 in tomato bacterial wilt were determined by comparative analysis with *Bacillus licheniformis* DSM13 Δ*alsS* that did not produce 2,3-BDO and *B. licheniformis* DSM13 producing meso-2,3-BDO. Tomato seedlings treated with the *B. licheniformis* DSM13 and the *K. pneumoniae* JCK-2201 culture broth formed meso-2,3-BDO that significantly diminished *R. solanacearum*-induced disease harshness regarding the control values.

The various contributions to this Research Topic are evidence that the exploitation of food microbiology would allow embracing a sustainable agriculture, while warning that, at worst for now, some elected microorganisms could replace synthetic fertilizers or pesticides. We trust that the Research Topic appropriately informs readers about the benefits that nature offers to the field of agro-food microbiology and about the challenges that have to be overcome in this field yet.

## Author contributions

All authors listed have made a substantial, direct, and intellectual contribution to the work and approved it for publication.

## Conflict of interest

The authors declare that the research was conducted in the absence of any commercial or financial relationships that could be construed as a potential conflict of interest.

## Publisher's note

All claims expressed in this article are solely those of the authors and do not necessarily represent those of their affiliated organizations, or those of the publisher, the editors and the reviewers. Any product that may be evaluated in this article, or claim that may be made by its manufacturer, is not guaranteed or endorsed by the publisher.

